# BACE1, the Alzheimer's beta-secretase enzyme, in health and disease

**DOI:** 10.1186/1750-1326-7-S1-L3

**Published:** 2012-02-07

**Authors:** Robert Vassar

**Affiliations:** 1 Northwestern University, Chicago, IL USA

## Background

The beta-secretase enzyme BACE1 initiates the production of beta-amyloid (Abeta), the presumptive cause of Alzheimer’s disease (AD). Recent promising advances have been made toward the identification of BACE1 inhibitor drugs. However, the development of BACE1 drugs has also proven challenging. Other targets upstream of BACE1 responsible for the regulation of enzyme levels and activity should be identified and evaluated, as they may offer alternative or complementary therapeutic strategies to direct BACE1 inhibition. Of particular relevance in this regard is the observation that BACE1 levels are elevated in AD brain, suggesting increased BACE1 levels may play a role in AD pathogenesis. If so, normalizing BACE1 levels may prove therapeutically efficacious while still allowing normal BACE1 function. Interestingly, impaired glucose metabolism occurs early in AD, and our work has demonstrated that glucose deprivation increases BACE1 levels and Abeta production in the brains of an APP transgenic model of AD, the Tg2576 mouse. We have identified the molecular mechanism of the BACE1 increase and showed that glucose deprivation induces phosphorylation of the translation initiation factor eIF2alpha (eIF2a-P), which in turn increases the translation of BACE1.

## Methods

BACE1-overexpressing 293 cells, primary neuron cultures, APP transgenic mice, and AD brain samples were analyzed by western blot for BACE1, eIF2alpha, and other markers. Genetic and pharmacological manipulation of eIF2alpha phosphorylation was performed in vitro experiments. eIF2alpha S51A knock-in mice and GADD34-AAV injection into mouse brain was used for manipulation of eIF2alpha phosphorylation in vivo.

## Results

Pharmacologically inducing eIF2alpha phosphorylation directly increases BACE1 levels. Conversely, genetically preventing eIF2alpha phosphorylation blocks the glucose deprivation-induced BACE1 increase. Chronic glucose deprivation in Tg2576 mice increases levels of eIF2a-P, BACE1, Abeta, and amyloid plaques. Importantly, eIF2a-P and BACE1 are elevated in aggressive plaque-forming 5XFAD transgenic mice, and levels of BACE1, eIF2α-P, and amyloid are all correlated in humans with AD. Abeta itself also appears to increase eIF2a-P and BACE1 in vivo.

## Conclusion

These results strongly suggest that eIF2alpha phosphorylation increases BACE1 levels and causes Abeta overproduction, which could be an early, initiating molecular mechanism in sporadic AD. Taken together, our work is consistent with the hypothesis that impaired glucose metabolism may lead to increased BACE1 levels by the eIF2a-P translational mechanism, and subsequent elevation of Abeta generation. Once amyloid plaques form, Abeta causes BACE1 levels to increase further, thus accelerating Abeta generation and plaque growth via a positive-feedback loop.

